# Elucidating structural variability in p53 conformers using combinatorial refinement strategies and molecular dynamics

**DOI:** 10.1080/15384047.2023.2290732

**Published:** 2023-12-10

**Authors:** Deborah F. Kelly, Maria J. Solares, William J. Dearnaley

**Affiliations:** a Department of Biomedical Engineering, Pennsylvania State University, University Park, PA, USA; b Center for Structural Oncology, Pennsylvania State University, University Park, PA, USA; c Molecular, Cellular, and Integrative Biosciences Graduate Program, Huck Institutes of the Life Sciences, Pennsylvania State University, University Park, PA, USA

**Keywords:** Tumor suppressor protein, p53, real-space refinement, simulated annealing, molecular dynamics, cryo-electron microscopy (EM)

## Abstract

Low molecular weight proteins and protein assemblies can now be investigated using cryo-electron microscopy (EM) as a complement to traditional structural biology techniques. It is important, however, to not lose sight of the dynamic information inherent in macromolecules that give rise to their exquisite functionality. As computational methods continue to advance the field of biomedical imaging, so must strategies to resolve the minute details of disease-related entities. Here, we employed combinatorial modeling approaches to assess flexible properties among low molecular weight proteins (~100 kDa or less). Through a blend of rigid body refinement and simulated annealing, we determined new hidden conformations for wild type p53 monomer and dimer forms. Structures for both states converged to yield new conformers, each revealing good stereochemistry and dynamic information about the protein. Based on these insights, we identified fluid parts of p53 that complement the stable central core of the protein responsible for engaging DNA. Molecular dynamics simulations corroborated the modeling results and helped pinpoint the more flexible residues in wild type p53. Overall, the new computational methods may be used to shed light on other small protein features in a vast ensemble of structural data that cannot be easily delineated by other algorithms.

## Introduction

Recent innovations in high-resolution imaging and computational modeling have vividly expanded our view of cancer biology at the nanoscale. These advances are attributed to improvements in camera technology, electron microscope (EM) optics, and high-performance resources including software and hardware interfaces.^1–3^ Biomedical research capitalizes on high-resolution assessments to provide sub-nanometer views of whole cancer cells, multi-component complexes formed in disease models, and individually mutated proteins or nucleic acids.^4–6^ To elucidate attributes of dynamic biological entities and expand knowledge of their exquisite mobility, complementary approaches are needed to pair with nanoscale imaging information. As such, research aimed at dissecting conformational variability of macromolecular structures can broaden our insights of their inherent properties and mechanisms.

In the cryo-EM field, there is a growing niche for algorithm development to gauge molecular flexibility among heterogenous states of molecules.^7–9^ Small flexible proteins are particularly challenging to study as standard classification methods may not convey the full ensemble of dynamic regions.^10^ As multiple conformers may be hidden within the confines of an EM map, dissecting this information permits us to expand our interpretation of nuanced variations in structural data.^11^ Correspondingly, new models to interpret EM maps may be computed through automated means or iterative refinement protocols in conjunction with multiple rounds of validation measures.^8,9,12–18^

To further investigate structural variations among low molecular weight proteins, we utilized recent modeling data determined for wild type p53 protein structures derived from human cancer cells.^19,20^ These structures were selected due to their small mass (~50 kDa and ~ 100 kDa for monomer and dimer states, respectively) and their dynamic ability to interact with disparate binding partners and nucleotides. Consequently, mutated forms of p53 are notorious molecular culprits in human cancer, along with being potential drug targets for pharmaceutical development.^21–24^

Here, we evaluated recent p53 monomer and dimer structures using a computational framework that combines real-space refinement routines with simulated annealing and energy minimization steps.^25–27^ Results produced new and improved convergent states for the p53 monomer and dimer models. Using this systematic approach, convergent models were reproducibly calculated having similar model fit statistics for the different p53 states. Additional molecular dynamics simulations for each oligomer shed light on the range of motion and potential conformational variability for p53 in solution. As each domain of the protein contains flexible regions, new insights from this work help pinpoint mobile units in p53 that support its engagement with other binding partners or cancer-related functions.

## Results

### Evaluating new conformers of the p53 monomer

**Figure 1.**
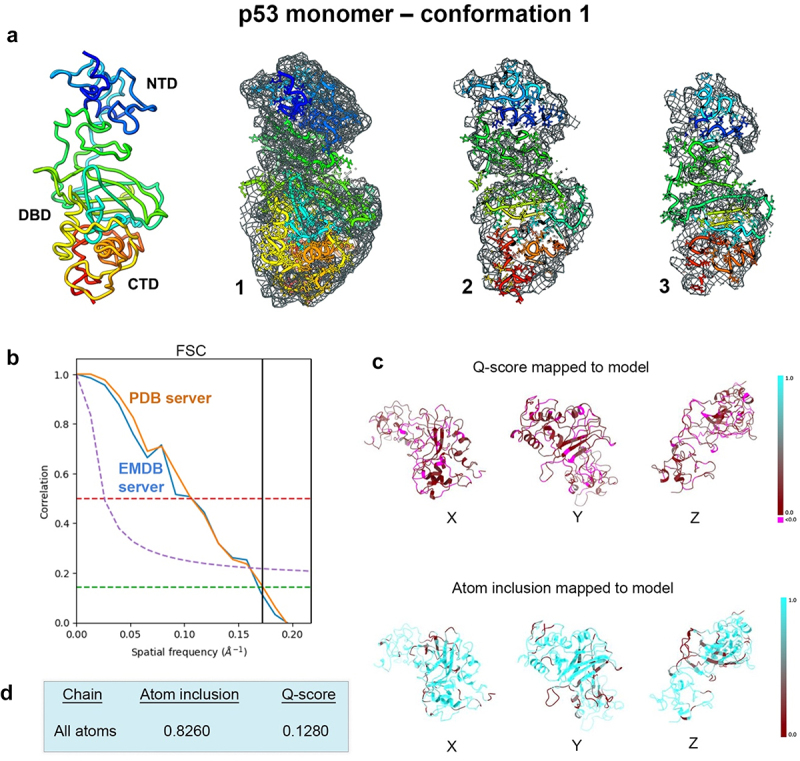
Conformation 1 determined for the p53 monomer. (a) the newly refined p53 structure had ~82% occupancy of the protein backbone and side chain residues within the EM map and slices through the map and model (1–3) highlight some of the fit residues. (b) Spatial resolution (~5.8-Å) was estimated at the FSC-0.143 value (green line) for half map comparisons employing the PDB validation server and the EMDB server. Both curves are shown for comparison. (c, d) the Q-score and atom inclusion values were 0.1280 and 0.8260 and are shown mapped to the model with scale values ranging from 0.0 (red) to 1.0 (cyan).

**Figure 2.**
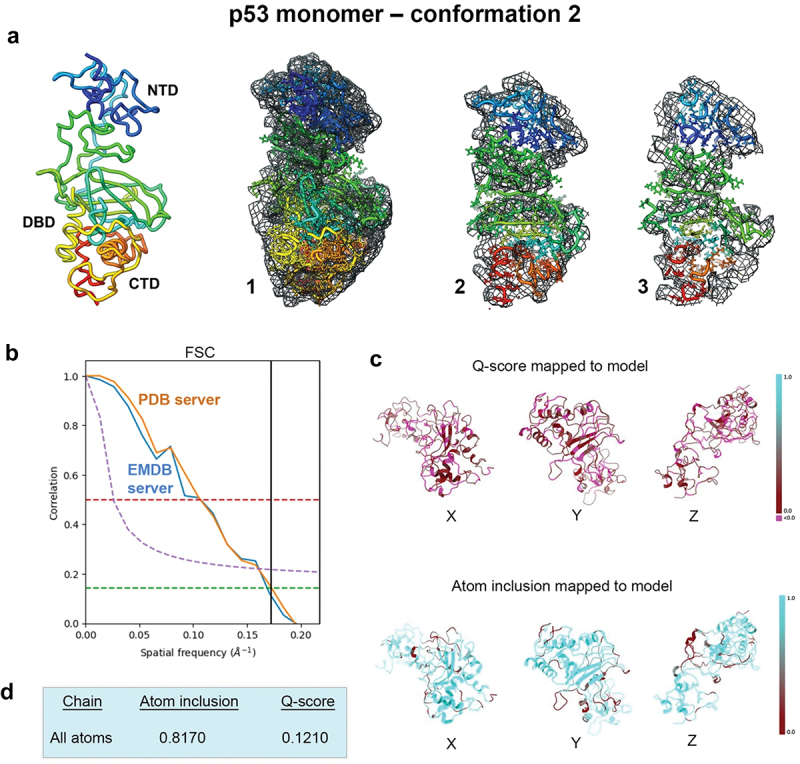
Evaluating conformation 2 determined for the p53 monomer. (a) the second refined model was fit in the EM map having ~81% occupancy of the c-α backbone and side chain residues, and slices through the map and model (1–3) highlight some of the fit residues. (b) Spatial resolution (~5.8-Å) was estimated according to the FSC-0.143 value (green line) calculated using the PDB validation server and the EMDB server. (c, d) the Q-score and atom inclusion values were 0.1210 and 0.8170, respectively, and are shown mapped to the model with scale values ranging from 0.0 (red) to 1.0 (cyan).

We performed a modeling analysis using prior data determined for the p53 monomer structure (EMD-28817; PDB, 8F2I)^20^ The main goal was to better understand how conformational variability can influence the overall p53 backbone. The EM map was imported into the PHENIX software package and auto-boxed upon import.^25,26^ This boxing procedure was an integrated step in the auto-sharpening routine. As a result, the auto-box process yielded a rectangular box around the center of the map, rendering it no longer cuboidal. The map was auto-sharpened with a resolution limit of 6.0-Å, in accordance with half-map resolution validation measurements. The p53 monomer model was imported into PHENIX at the same origin as the EM map. The model was subjected to iterative rounds of real space refinement routines using standard procedures in the program workflow. Two conformations emerged upon convergence and were further evaluated for stereochemistry and refinement statistics (Figures 1, 2, Table 1).

**Table 1.** Model refinement and validation.

	Conformation 1 (dimer)	Conformation 2 (dimer)	Conformation 1 (monomer)	Conformation 2 (monomer)
Map parameters				
Symmetry group	C2	C2	C1	C1
Pixel size (Å/pixel)	1.2	1.2	2.3	2.3
Map resolution (Å); FSC-0.143	4.4	4.4	5.8	5.8
Model refinement				
Refinement software	PHENIX	PHENIX	PHENIX	PHENIX
Refinement strategies	Rigid body, simulated annealing, energy minimization	Rigid body, simulated annealing, energy minimization	Rigid body, simulated annealing, energy minimization	Rigid body, simulated annealing, energy minimization
Chains	A, B	A, B	A	A
Residues	391/393 (A) 391/393 (B)	391/393 (A) 391/393 (B)	391/393	393
Resolution cutoff	4 Å	4 Å	5 Å	5 Å
Map-to-model (masked/unmasked)				
Map alone (d99) (Å)	5.25/5.26	5.25/5.26	5.13/5.14	5.13/5.14
Overall B iso	130/130	135/135	305/325	330/355
d_model (Å)	5.10/5.10	5.10/5.10	4.80/4.80	4.80/4.80
FSC (model) 0.143 (Å)	4.87/4.97	4.90/4.99	5.06/5.08	5.08/5.10
Q-score	0.1790	0.1730	0.1280	0.1210
CC_mask	0.3195	0.3213	0.5167	0.5022
CC_volume	0.3368	0.3324	0.5325	0.5166
Model validation				
MolProbity	2.27	2.12	2.25	2.22
Clashscore	14	11	16	14
Ramachandran				
Favored (%) A, B	88	90	91	90
Allowed (%)	12	10	9	10
Outliers (%)	0	0	0	0
Rotamer outliers (%)	0	0	0	0
C-beta deviations	0	0	1	0
Bad bonds	2/6286	0/6286	0/3143	1/3143
Bad angles	6/8542	2/8542	0/4272	0/4272

The first new conformation of the p53 monomer was calculated using rigid body refinement with integrated simulated annealing and energy minimization routines. The output model had ~ 80% occupancy of the c-α backbone and side chain residues and fit well within the map (Figure 1a). Spatial resolution (~5.8-Å) was estimated at the FSC-0.143 value employing the Protein Data Bank (PDB) validation server and the Electron Microscopy Data Bank (EMDB) Fourier Shell Correlation (FSC) server. Both curves are shown for comparison in Figure 1b. Map-model resolvability (Q-score)^12,13^ and atom inclusion values were 0.1280 and 0.8260 respectively (Figure 1c,d). Additional output from PHENIX for model-to-map fit cross-correlation included values for CC_mask (0.5167) and CC_volume (0.5325). The overall MolProbity^28,29^ score for flexible conformation 1 was 2.25 and no Ramachandran outliers were identified (Table 1).

A second closely related model emerged from the same computational procedures that we referred to as conformation 2. Rigid body refinement, simulated annealing and energy minimization steps were again implemented in PHENIX using standard protocols. The second p53 monomer model showed similar occupancy of the c-α backbone and side chain residues within the same EM map (Figure 2a).

The spatial resolution estimates are again highlighted in Figure 2b. The Q-score (0.1210) and atom inclusion value (0.8170) were slightly lower for the second p53 monomer model than for the first model. Model to map fit cross-correlation output from PHENIX included CC_mask (0.5022) and CC_volume (0.5166). The MolProbity score for flexible conformation 2 was 2.22, which was similar to the first conformation. No Ramachandran outliers were identified and the clashscore for flexible conformation 2 was ~ 14, an improvement over flexible conformation 1, which was ~ 16 (Table 1).

**Figure 3.**
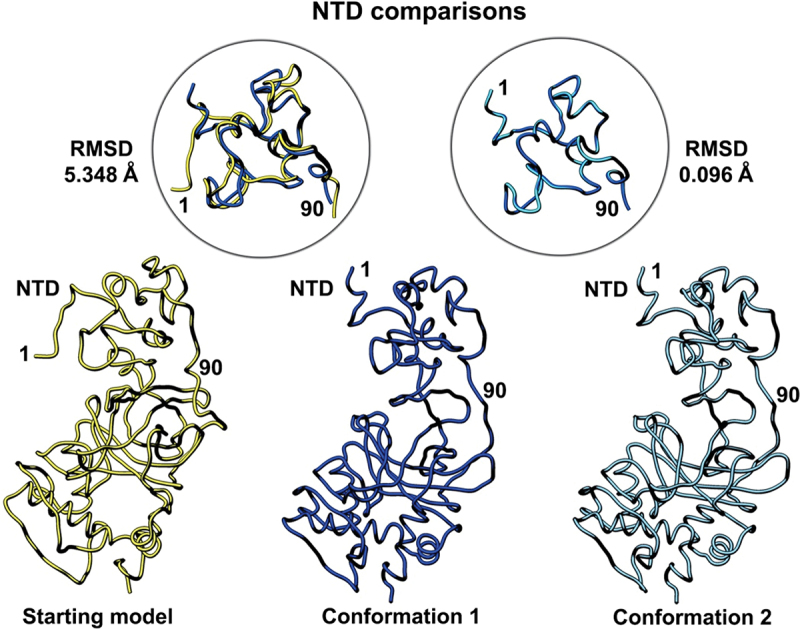
Side-by-side comparison of the refined p53 monomer models focusing on the N-terminal domain (NTD). Model alignments were evaluated for the starting model (PDB code, 8F2I, yellow) with respect to the two new refined models using the Chimera program and structure comparison tools. Differences in c-alpha backbones were quantified using RMSD values. Conformation 1 is in dark blue while conformation 2 is colored light blue.

**Figure 4.**
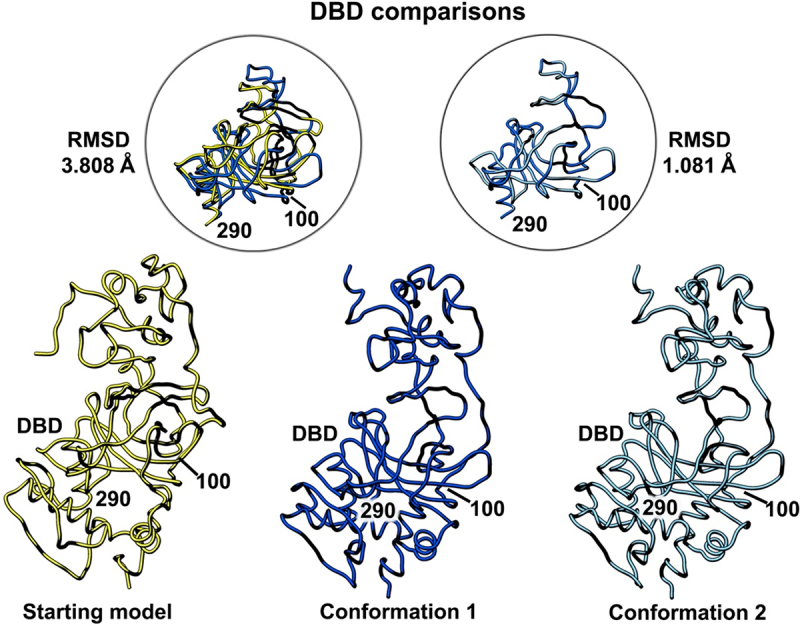
Side-by-side comparison of the refined p53 monomer models highlighting the DNA binding domain (DBD). Model alignments were evaluated for the starting model (PDB code, 8F2I, yellow) with respect to the two newly refined models using the Chimera program and structure comparison tools. Differences in c-alpha backbones were quantified using RMSD values. Conformation 1 is in dark blue while conformation 2 is colored light blue.

**Figure 5.**
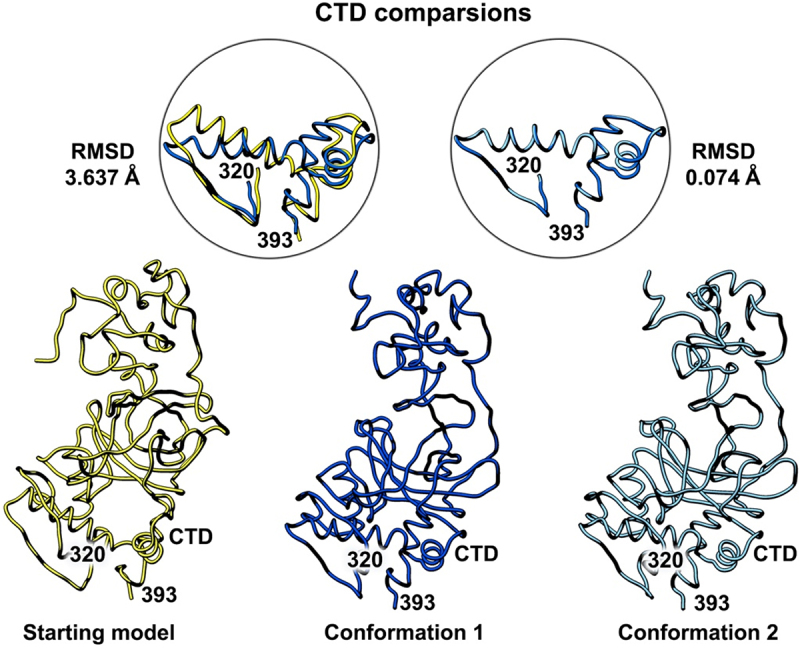
Side-by-side comparison of the refined p53 monomer models emphasizing the C-terminal domain (CTD). Model alignments were evaluated for the starting model (PDB code, 8F2I, yellow) with respect to the two new refined models using the Chimera program and the structure comparison tools. Differences in c-alpha backbones were quantified using RMSD values. Conformation 1 is in dark blue while conformation 2 is colored light blue.

To better understand how the newly refined models compare with the original model deposition (pdb code, 8F2I),^20^ we performed a side-by-side comparison displayed in Figures 3–5. Models were aligned using structure comparison tools in the Chimera software package.^30^ Each structural domain of interest is shown separately for ease of comparison. Firstly, the N-terminal domain (NTD) of the p53 monomer (residues 1–90) showed the highest degree of flexibility as it generally lacks secondary structure. A comparison of the root-mean-squared deviation (RMSD) for the starting model (PDB code, 8F2I, yellow) and conformation 1 (dark blue) was 5.348 Å. A comparison of conformation 1 and conformation 2 (light blue) in the NTD yielded an RMSD value of 0.096 Å. These quantitative comparisons indicate some flexibility between the deposited model and the newly refined models.

A similar comparison of the DNA binding domain (DBD, residues 100–290) showed less flexibility than the NTD region as there is one helix present in this domain along with a few beta strands (Figure 4). The presence of more well-defined structural elements generally indicates greater protein stability in comparison to flexible loop regions. The RMSD comparison for the starting model (PDB code, 8F2I, yellow) and conformation 1 (dark blue) was 3.808 Å. A comparison of conformation 1 and conformation 2 (light blue) in this region yielded an RMSD value of 1.081 Å. These quantitative values indicate only minor differences among all three models.

A final comparison of the C-terminal domain (CTD) comprised of residues 320–393 showed the fewest differences and there is one helix present in this domain. It is important to note this segment is shorter than the other two functional regions of the protein (Figure 5). The RMSD comparison for the starting model (PDB code, 8F2I, yellow) and conformation 1 (dark blue) was 3.637 Å. A comparison of conformation 1 and conformation 2 (light blue) for the CTD yielded an RMSD value of 0.074 Å. These quantitative values indicate minor differences between the deposited model and the new models.

### Conformational dynamics of p53 monomer models

**Figure 6.**
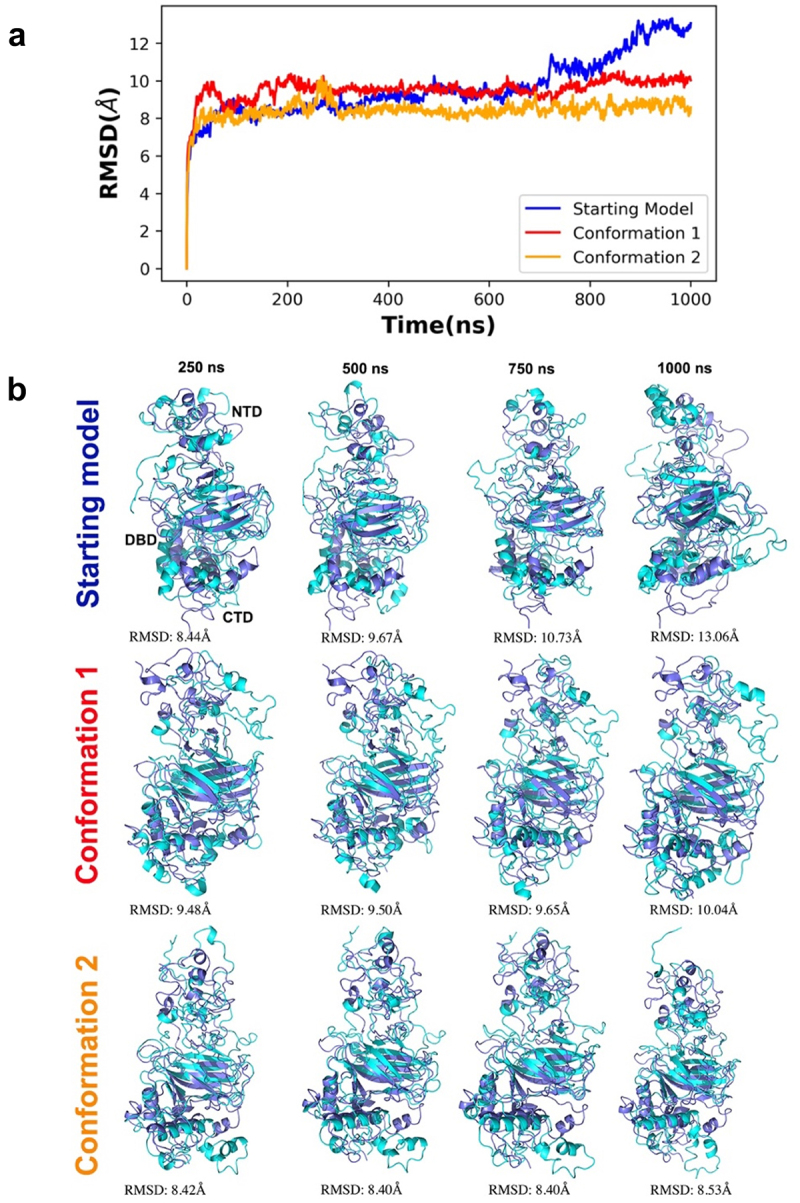
Time series RMSD analysis of simulated trajectories for p53 monomers. (a) the different model conformations were plotted over 1000 ns with the starting model in blue, conformation 1 in red, and conformation 2 in orange. Lower RMSD values indicate a lower deviation from the starting model (reference structure) whereas higher RMSD values indicates a higher deviation of the conformers generated throughout the simulation. (b) superimposition of simulated snapshots (cyan) with their respective reference structures (purple) show domain displacements, particularly in N terminal region. The first row illustrates the superimposition of the starting model and its simulated conformers. The second row illustrates the superimposition of conformation 1 and its simulated conformers while the third row illustrates the superimposition of conformation 2 and its simulated conformers.

To better understand the conformational variability and dynamics of the different p53 monomeric models, one microsecond all-atom molecular dynamics (MD) simulations were performed (**Movie S1**). We compared the starting reference model with conformation 1 and conformation 2 by calculating the RMSD of the C-α backbone from each simulation trajectory (Figure 6a). We first attempted pairwise RMSD clustering, but the resolution of the clustering graph did not permit us to extract usable features for conformational assessments. Thus, we manually superimposed snapshots extracted at different time points from the simulated trajectories in comparison to the reference structure. These results showed that the central part of p53 that contains the DBD is the most stable region among all three models through the simulations. By contrast, the NTD and CTD showed the largest differences among conformers, with more pronounced changes in the CTD for the reference structure than for conformations 1 and 2. Notably, the N terminal portion of conformation 2 was more stable than the starting reference and conformation 1. Conformation 2 did not undergo drastic transitional changes compared to the other models and its trajectories were more stable and well converged compared to the starting model (Figure 6b).

**Figure 7.**
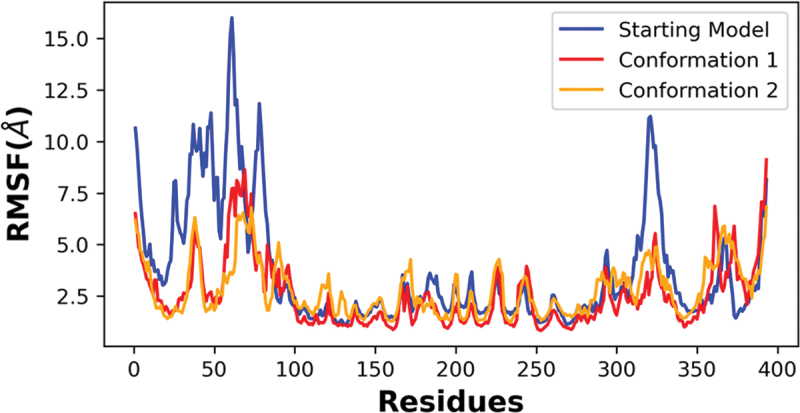
RMSF analysis of the alpha carbon atoms for p53 monomers. A comparison of the different p53 monomer conformations is plotted across all c-alpha atoms with the starting model in blue and conformations 1 and 2 colored red and orange, respectively. Higher fluctuations of RMSF values indicates higher residual flexibility, whereas lower fluctuations of RMSF values indicates lower residual flexibility.

Simulations for each system were run two times on the same GPU host using a higher precision mode and no differences were observed, indicating high reproducibility of the simulation data. Random reference structures were not assigned for RMSD measurements as this procedure would not lead to representative conformers required for structural comparisons. The local residue flexibility analysis (RMSF) of the simulated trajectories showed that NTD of the starting model were more flexible than two newly refined models (Figure 7). There were also residues in the CTD of the starting model (320–340; 360–370) that varied in comparison to the new conformers. This quantitative analysis supports the side-by-side comparisons of the refined p53 monomer models with respect to the reference model as discussed earlier.

### Model convergence for the p53 dimer

**Figure 8.**
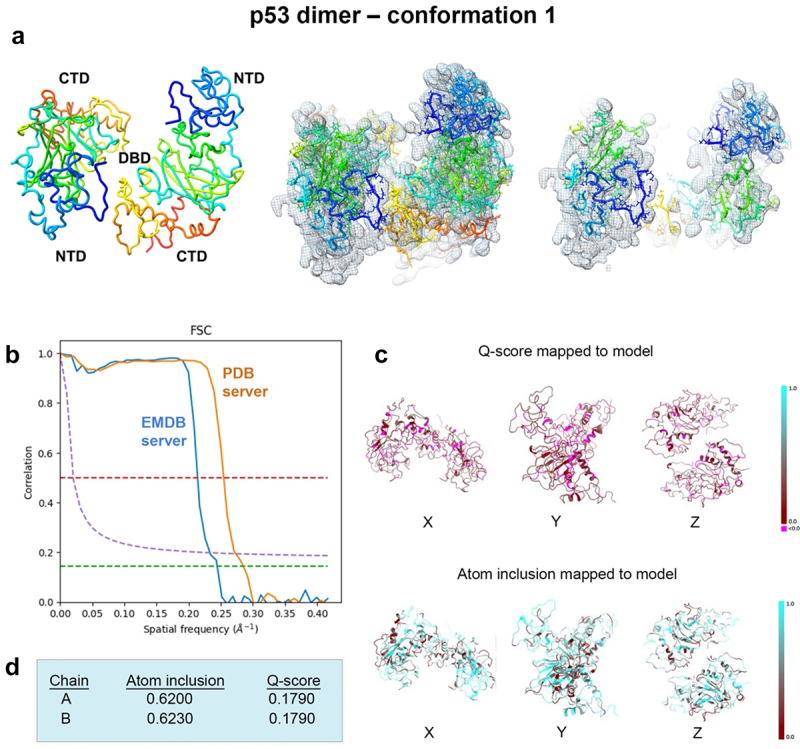
Conformation 1 of the refined p53 dimer model. (a) the refined model of the p53 dimer fit in the EM map. Slices through the map and model highlight some of the fit residues within the map. (**b**) Spatial resolution (~4.4-Å) was estimated at the FSC-0.143 value (green line) employing the EMDB FSC server. The PDB server slightly over-estimated the resolution. **(c,d)** Q-scores for chains A, B were 0.1790 and atom inclusion values were 0.6200 and 0.6230, respectively and shown mapped to the model. Scale ranges from 0.0 (red) to 1.0 (cyan).

**Figure 9.**
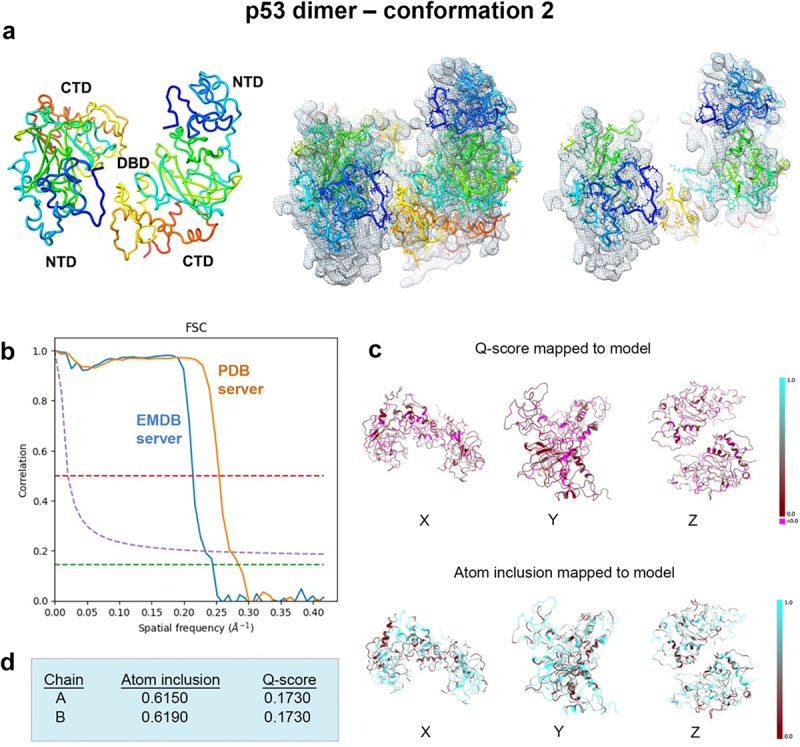
Conformation 2 of the refined p53 dimer model. (a) a second refined model of the p53 dimer is shown fit in the EM map and slices through complex highlight some of the fit residues in the map. (b) Spatial resolution (~4.4-Å) was estimated at the FSC-0.143 value (green line) for half map comparisons employing the EMDB FSC server. The PDB server slightly over-estimated the resolution. (c, d) Q-scores for chains A, B were 0.1730 and atom inclusion values were 0.6150 and 0.6190, respectively and shown mapped to the model. Scale ranges from 0.0 (red) to 1.0 (cyan).

To improve knowledge of conformational nuances in the p53 dimer, we performed a similar analysis using recent EM modeling data (EMD-28816; PDB, 8F2H).^20^ As the p53 dimer (~100 kDa) is larger than the monomer (~50 kDa) structural features were more clearly defined within the density. The map was imported into the PHENIX software package and auto-boxed while implementing the auto-sharpening routine at a resolution limit of 4.5-Å. Upon implementing the auto-box routine, a rectangular box segmented the map around its center rendering it no longer cuboidal. The corresponding p53 dimer model (pdb code, 8F2H) was imported into the PHENIX package at the same origin as the EM map and subjected to iterative real-space refinement routines. Two new models converged for the p53 dimer that fit well within the map (Figures 8, 9). Model refinement statistics are provided in Table 1. The p53 dimer models were subjected to multiple rounds of rigid body refinement along with simulated annealing and energy minimization (Figure 8a).

Spatial resolution (~4.4 Å) was determined at the FSC-0.143 value (Figure 8b) employing EMDB FSC server while the PDB validation server slightly over-estimated the resolution value. The overall Q-score and atom inclusion values for chains A and B were 0.1790 and 0.6200 and 0.6230, respectively (Figure 8c,d). Model to map fit cross-correlation output from PHENIX included: CC_mask (0.3195) and CC_volume (0.3368). The overall MolProbity score for conformation 1 was 2.27, no Ramachandran outliers were identified, and the all-atom clashscore was ~ 14 (Table 1).

A second closely related model, conformation 2, also emerged using the same real-space refinement procedures (Figure 9a). The resolution estimate of the map is highlighted in Figure 9b. The Q-score (0.1730) and atom inclusion values (chain A, 0.6150; chain B, 0.6190) were slightly lower for model 2 than for model 1. Model to map fit cross-correlation values from PHENIX included CC_mask (0.3212) and CC_volume (0.3324). These values were similar for conformation 2 in comparison to conformation 1. The MolProbity score for conformation 2 was 2.12, similar to conformation 1 (2.27), and no Ramachandran outliers were identified (Table 1). The all-atom clashscore was ~ 11, which is slightly better than for conformation 1.

**Figure 10.**
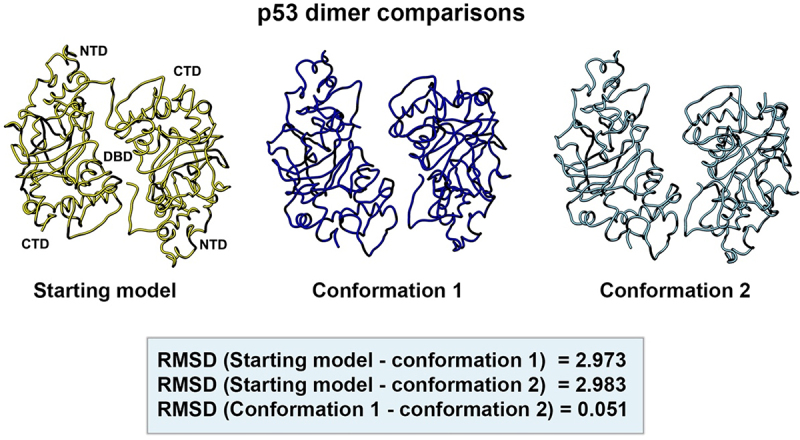
Side-by-side comparison of the refined p53 dimer models. Model alignments were evaluated for the starting model (PDB code, 8F2H, yellow) and the two new refined models for p53. Differences in the protein backbones were quantified by RMSD values. Conformations 1 (dark blue) and 2 (light blue) were most similar with minor backbone differences noted between the reference and the two new conformers.

A side-by-side comparison of the two new models with respect to the original starting model (PDB code, 8F2H) is shown in Figure 10. The models were aligned in the Chimera software package using structure comparison tools. For the starting model (yellow) and conformation 1 (dark blue) the RMSD value was 2.973 Å. A comparison of the starting model and conformation 2 (light blue) yielded an RMSD value of 2.983 Å. The difference between the two new refined models produced an RMSD value of 0.051 Å. These quantitative values indicate a low degree of variation among the three structures.

### Conformational dynamics of p53 dimer model

**Figure 11.**
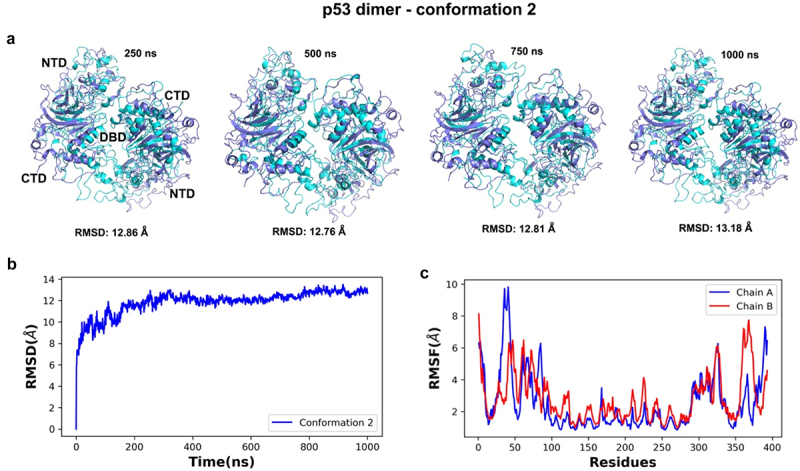
Time series and dynamic analyses of the simulated trajectory for conformation 2 of the p53 dimer. (a) superimposition of simulated snapshots (cyan) with the reference structure (purple) for conformer 2 showed residue displacements in *N*- and the C-terminal domains of both protein chains. (b) lower RMSD values after 400 ns of simulation indicate a lower deviation from the starting conformer after that timeframe and more stability throughout the simulation. (c) the RMSF analysis of alpha carbon atoms for the p53 dimers were plotted across all c-alpha atoms. Higher fluctuations were noted in the NTD and CTD (chains a and B) than in the central DBD regions of the dimer.

Due to computational requirements to simulate three dimer models for one microsecond, three short simulations (100 ns) were alternatively computed for each model. We chose conformation 2 for extending the simulation time to one microsecond (**Movie S2**) based on initial structural insights. The RMSD analysis of the simulated trajectory showed that conformation 2 was stable and no significant RMSD deviations were seen after the 400 ns of simulation, meaning there was lower variability after this timeframe (Figure 11a). RMSD values suggested the DBD was the most stable region throughout the p53 models with larger fluctuations occurring among residues 30–50 (NTD), 300–330 (CTD), and 360–380 (CTD) for chains A and B (Figure 11b). Interestingly, the dimer model showed about the same degree of flexibility in the N terminal region as the monomer model, but the C-terminal domain in the dimer showed greater flexibility than the monomer models (compare Figure 6, 7 and Figure 11). This flexibility in the dimer CTD may be related to its function in regulating the physical accessibility of the DBD when triggered for DNA repair. Additional experimental work is in progress to test this idea under cellular stress conditions.

## Discussion

Computational analyses were conducted on the p53 protein models using real space refinement procedures executed as part of the standard workflow in the PHENIX software package.^25,26^ Two closely related conformations were identified for the p53 dimer and monomer states during these evaluations. Each new model fit well within the EM maps and quantitative methods validated the quality of the modeling results. In each instance, improved map-to-model agreement was achieved using rigid body refinement routines that incorporated simulated annealing and energy minimization steps. As part of this procedure, we closely monitored for close contacts, which did not present a major issue during refinement in the PHENIX software package.

A direct comparison between the new p53 dimer models and the deposited model (pdb code, 8F2H) revealed minor overall differences in RMSD values for each model. Greater variations are often seen in NMR structures used to interpret structural models of individual protein domains. Similarly, in the case of the p53 monomer, differences in each functional domain varied from ~ 3–5 Å, also indicating only minor changes in the backbone positions in the newly refined models compared with the starting model (pdb code, 8F2I). Despite these nuanced changes in the protein models, the new statistics for the model fitting were much improved in the refined p53 dimer and monomer cases. This new information can be used moving forward to ensure additional checks and balances in model fitting for future studies.

It is also important to note, that the native p53 proteins were isolated from human cancer cells using well-established cell lines (U87-MG line). Years of recombinant protein expression of p53 have provided limited structural information for full-length p53 and have primarily focused on the DBD.^31–33^ While the current work with natively sourced proteins is making good progress, the approach does present certain challenges. For example, post-translational modifications are prevalent in the p53 protein, which can blur map features during image processing routines, and the maps presented here likely represent a heterogenous mixture of these modified forms.

Overall, the new p53 monomer and dimer models showed some flexibility following real-space refinement techniques performed using intermediate resolution EM maps in the range of 4–6 Å. This level of analysis, however, may enable future investigations of larger p53 assemblies interacting with genomic DNA. Equally important, different isoforms of p53 can exist simultaneously in human cells, and these variations of the protein are difficult to parse out using structural techniques. Current efforts are underway to better define modifications and isoforms in native samples using mass spectrometry and other biochemical techniques. In general, these variables can influence features in the EM maps and the accuracy of model fitting. They can also account for differences in map-to-model assessments observed for the natively sourced proteins rather than those from recombinant sources.

While p53 has intrinsically disordered segments in the NTD and parts of the CTD, it does exhibit inherent stability in the central region of the protein. The stability of the central DBD was noted in both monomer and dimer forms of p53 and this finding is consistent with the work of Chillemi et al, 2013.^34^ In the prior study, the team developed a theoretical model for the full-length p53 monomer and conducted elegant MD simulations, resulting in reliable RMSD and RMSF measurements. The RMSF plots calculated from our experimental models have very similar profiles to those calculated for the theoretical models in the prior work. Only a few shifts were noted in fluctuation values within the CTD for the full-length monomer structure. While the architecture of the Chillemi model shows some differences in comparison to our experimental model, the MD results are generally consistent between the two studies in terms of protein stability insights.

Our long-term goal is to assess the impact of p53 mutations on its structure and function across a variety of cancers. In this regard, the presented methods lay a strong foundation to study conformational flexibility among different isoforms and oligomeric states with broad potential applications for other proteins. Although the current analysis was established for p53 monomer and dimer structures, a natural extension of the work is to focus on dynamic properties of p53 tetramers, which primarily contact DNA during repair events. As new models become available for full-length p53 tetramers and other isoforms, molecular perturbations can be reasonably assessed with respect to wild type structures using MD simulations and variability mapping. This information may be used in future applications to shed light on p53 missense mutations and their contribution to cancer induction.

## Materials and methods

### Protein sources

Native, wild type p53 proteins and assemblies were isolated from U87MG cells that are commercially available from the American Type Culture Collections (ATCC). Protocols used to isolate the native proteins have been demonstrated and fully described in prior work.^19,20^

### EM data and initial modeling

Specimen preparation methods, EM data collection parameters, and initial modeling procedures are previously described.^19,20^ A summary of map parameters is included in Table 1.

### Real-space refinement routines

EM maps and model coordinates for the p53 monomer (EMD-28817; PDB, 8F2I) and dimer structures (EMD-28816; PDB 8F2H) were analyzed. The p53 monomer map was imported into the PHENIX software package^25,26^ at a sampling value of 2.3 Å/pixel and auto-boxed during import as part of the auto-sharpening procedure. The auto-box routine produced a rectangular box size around the center of the map, rendering it no longer cuboidal. A resolution cutoff of 5-Å was applied to the maps used for model refinement. The p53 monomer model was fit into the EM map in PHENIX and subjected to 5 macrocycles of rigid body refinement with simulated annealing. Iterative rounds of refinement and energy minimization were implemented until convergence. Likewise, the p53 dimer map was imported into PHENIX at a sampling value of 1.2 Å/pixel. The map was auto-boxed during import as part of the auto-sharpening procedure, and a resolution cutoff of 4.5-Å was applied to the maps that were used for subsequent model refinement. The p53 dimer model was subjected to 5 macrocycles of rigid body refinement with simulated annealing. Iterative rounds of rigid body refinement and energy minimization were implemented until convergence. Q-scores and atom inclusion values were calculated using the PDB validation server.

### Model alignment and superimposition procedures

Flexible models of the p53 monomer were aligned using structure comparison tools implemented in the Chimera software package.^30^ Root mean square deviation (RMSD) measurements were calculated using the Morph Conformations function in Chimera. The p53 reference model was held in place while the test model was superimposed upon the reference. RMSD values were output by the Chimera program. The same alignment and superimposition procedures were implemented in the Chimera program to compare the p53 dimer models.

### Molecular dynamics simulation

One microsecond molecular dynamics simulations were performed using Desmond module of Schrodinger suite (Schrödinger Release 2022–4: Desmond Molecular Dynamics System, D. E. Shaw Research). We employed the previously published wild type structures of the p53 monomers and dimers and newly refined structures of p53 for system building and MD simulations. Prior to MD simulations, the three-dimensional structures of P53 was subjected to preprocessing. The protonation states of residues were generated and optimized through the determination of pKa by PROPKA.^35^ The structure was then energy minimized and considered for system building. The TIP3P water model was used for solvating the system.^36^ The OPLS3e force field was used for parametrizing protein coordinates.^37^ A cubic box boundary with 10 angstrom buffer distance was chosen for defining and calculating the simulation box size. The net charge of the system was calculated and neutralized by adding 3 Na+ ions. 0.15 M NaCl was added to the system as counterions and mimicking the ionic strength of the environment, which helps to stabilize the structure and reduce the effects of long-range interactions. Prior to final the MD production, the system was simulated in the Berendsen NVT ensemble at a temperature of 10 K to constrain the heavy atoms of the solute as well as prevent them from moving too quickly and causing the simulation to become unstable. The simulation was then switched to the NPT ensemble at a temperature of 310 K, 1 atm pressure, and thermostat relaxation time of 2 ps. The RESPA integrator was used with a time step of 2.0 fs. The Nose–Hoover thermostat^38^ and the Martyna–Tobias–Klein barostat were used to maintain the pressure and temperature at 310 K and 1 atm. For calculating short-range columbic interactions, a cutoff radius of 9 angstroms was used. The simulation snapshot was saved at 1 ns interval for trajectory analysis. The simulation interaction diagram module of Desmond and PyMol (The PyMOL Molecular Graphics System, Version 2.5.5) Schrödinger, LLC. was used for analyzing the simulation trajectory.

## Supplementary Material

Links to supporting movies.docx

## Data Availability

Molecular structures related to this work have been deposited in the Protein Databank and will be released after publication. Additional data may be made available upon request at the discretion of the Principal Investigator.
