# Correction

**DOI:** 10.1080/15384047.2024.2318833

**Published:** 2024-02-13

**Article title**: Elucidating structural variability in p53 conformers using combinatorial refinement strategies and molecular dynamics

**Authors**: Deborah F. Kelly, Maria J. Solares, and William J. Dearnaley

**Journal**: *Cancer Biology & Therapy*

**DOI**: https://doi.org/10.1080/15384047.2023.2290732

The author, Md Rimon Parves, withdraws name from the author list of the original article and hence the author list has been updated to Deborah F. Kelly, Maria J. Solares, and William J. Dearnaley.

