# Supplementary material for: Elucidating structural variability in p53 conformers using combinatorial refinement strategies and molecular dynamics
Source: Cancer Biol Ther. 2023 Dec 10;25(1):2290732. doi: 10.1080/15384047.2023.2290732 (PMC10732606; doi:10.1080/15384047.2023.2290732)
Supplement: Links to supporting movies.docx [file KCBT_A_2290732_SM3431.docx]

During the manuscript submission process, the editorial assistant asked that we upload the associated supplementary movies online and provide links to the files. Below are Dropbox links to view Movies S1 and S2:

**Movie S1**: https://[www.dropbox.com/scl/fi/dhw6iar31gdkaq38ixvs3/MovieS1.mov?rlkey=8cuaovvo8](http://www.dropbox.com/scl/fi/dhw6iar31gdkaq38ixvs3/MovieS1.mov?rlkey=8cuaovvo8) 8b46v6w7ymgbsvlm&dl=0

**Movie S2**: https://[www.dropbox.com/scl/fi/uqxjt68g9xpdrz6b8ywhx/MovieS2.mov?rlkey=bfgsow2ht](http://www.dropbox.com/scl/fi/uqxjt68g9xpdrz6b8ywhx/MovieS2.mov?rlkey=bfgsow2ht) bgo8n5ltefa3wi7i&dl=0
